# WTI, Brent or implied volatility index: Perspective of volatility spillover from oil market to Chinese stock market

**DOI:** 10.1371/journal.pone.0302131

**Published:** 2024-04-25

**Authors:** Peng Qin, Manying Bai

**Affiliations:** Department of Economics and Management, Beihang University, Beijing, P. R. China; Massey University - Albany Campus: Massey University - Auckland Campus, NEW ZEALAND

## Abstract

This study investigates the impact of oil market uncertainty on the volatility of Chinese sector indexes. We utilize commonly used realized volatility of WTI and Brent oil price along with the CBOE crude oil volatility index (OVX) to embody the oil market uncertainty. Based on the sample span from Mar 16, 2011 to Dec 31, 2019, this study utilizes vector autoregression (VAR) model to derive the impacts of the three different uncertainty indicators on Chinese stock volatilities. The empirical results show, for all sectors, the impact of OVX on sectors volatilities are more economically and statistically significant than that of realized volatility of both WTI and Brent oil prices, especially after the Chinese refined oil pricing reform of March 27, 2013. That implies OVX is more informative than traditional WTI and Brent oil prices with respect to volatility spillover from oil market to Chinese stock market. This study could provide some important implications for the participants in Chinese stock market.

## 1. Introduction

The energy-stock market relationship is a well-known important topic. The relationship may be dynamic due to some events and some researches focus on that dynamic spillover effects. For example, [1] employ a graph theory approach to explore the dynamic volatility transmission among US equities, strategic commodities (oil and gold) and BRICS equities. Based on VAR-GARCH model, the work by [2] try to disclose the dynamic linkage and spillover among carbon emission trading (CET) market, coal market and new energy stock market. The work of [3] investigate the dynamic volatility spillover between Chinese stock market and nine Chinese commodities through volatility spillover index derived from TVP-VAR. [4] investigate the dynamic volatility spillover between crude oil, new energy and resources related sectors. The energy-stock relationship may also differ in different market states. For instance, conditional on quantile-based method, [5] aim to describe volatility transmission among global equities, strategic commodities and the US Treasury bond market during normal and high volatility states. Note that there exist not only return and volatility spillover between energy market and stock market, higher moments namely skewness and kurtosis spillover may also contain valuable information. Based on realized estimators of return distributions across US stock, crude oil and gold markets, the work by [6] point out although realized volatility spillover effects is relatively stronger, spillovers in skewness, kurtosis and jumps are notable.

With the growing oil demand of China, it is interesting and beneficial for scholars and investors to investigate the oil-stock nexus between oil market and Chinese stock market. Some studies have paid attention to study how oil market affect the returns of Chinese stock market ([7–12]; among others). Another strand of literatures pay attention to the impact of oil market on the volatility of Chinese stock market [13–16]. Although the topic of this study is the impact of oil market on the other (Chinese stock market), oil market as a receiver rather than a giver attracts attentions either. For example, [17] show evidence in support of global economic activity as a good predictor of energy market volatility. With respect to high moment spillover, based on daily realized data of Chinese stock market index and eight Chinese commodity futures, [18] derive similar conclusions with [6]. Moreover, through the combination of quantile VAR model and time-varying parameter vector autoregressive (TVP-VAR) model, [19] further investigate the realized high-moments spillover among crude oil, gold, economic policy uncertainty (EPU) and four Chinese financial sectors including bank, trust, insurance and security under different market conditions. Most researches mentioned above pick the information derived from WTI oil price and/or Brent oil price to proxy for oil market information. However, [20] points out that crude oil implied volatility index (OVX) contains both historical and future information about oil market, which makes OVX seems more informative than raw historical oil prices. Since May 10, 2007, the Chicago Board Options Exchange (CBOE) lunched a new implied volatility index (OVX) in the crude oil market; the index, which is similar with VIX in stock market, is calculated conditional on options on the United States Oil Fund and can measure the market’s expectation of the 30-day volatility of crude oil prices. Moreover, [21] provide evidences that the impact of OVX on Chinese stock market returns is more significant than the realized variance of WTI oil price, especially after 2008 finance crisis. Specifically, they aim to investigate the impacts of oil price uncertainty on one Chinese stock composite index return and five Chinese sector returns. By using vector autoregression model (VAR), based on the sample from May 10, 2007 to December 31, 2015, they document the response of all six Chinese stock indexes returns to OVX shocks are statistically significant for both whole sample and subsample from June 31, 2009 to December 31, 2015 (post-crisis period). In line with the finds of [21,22] examine the impact of OVX change on the Chinese sector returns under different market conditions via quantile regression. They find significant impact for all 10 sectors, especially for the bear market conditions of China. More specifically, they find the OVX changes have significantly negative impacts on all 11 selected Chinese stock indices returns at low quantiles (0.05, 0.1, 0.25). Moreover, these negative effects are mainly caused the positive changes of OVX, which they call asymmetric impacts of OVX on Chinese stock returns.

Given the strong impact of OVX on Chinese stock returns and that market volatility investigation is also important, it is surprised that little attention was paid for the impact of OVX on the volatility of Chinese stock market. The one exception which seems related with this topic is [23], they investigate the impact of OVX change on the change of VXFXI. VXFXI is an implied volatility index reflects the market anticipation of Chinese stock market fluctuation, VXFXI is to Chinese stock market as VIX is to American stock market. It is firstly published by the CBOE on March 16, 2011. In their study, OVX change show significant positive impact on VXFXI change and the impact seems stronger in the bear market condition. Another related research is [24], through copulas and CoVaR methods, they investigate the extreme risk spillover between OVX, VXFXI and USD/CNY exchange rate market uncertainty (i.e., USDCNYV1M). The empirical evidences show the risk spillover effects from OVX to VXFXI is stronger than that from OVX to USDCNYV1M and that from USDCNYV1M to VXFXI.

[23,24] aim to investigate the impact of OVX on Chinese stock volatility, they choose OVX as the proxy of oil market uncertainty. Hence, they investigate the impact of only OVX on Chinese stock volatility and unlike them, this study concerns the impacts of three different oil market uncertainty indicators (OVX, WTI and Brent) and aims to identify which indictor should be the proxy of oil market uncertainty when focus on the volatility spillover from oil market to Chinese stock market. Moreover, they concentrate on the volatility of whole Chinese stock volatility while we investigate the impacts of oil market uncertainty indicators on 10 Chinese sector volatilities. Hence, this study mainly tries to answer two questions. Firstly, when focus on oil-Chinese stock volatility spillover, there are three widely-used benchmark indictors (OVX, WTI, Brent) to embody the oil market uncertainty while which indictor has the most influential impacts on Chinese stock volatility. Secondly, whether the strongest indictor is sector-specific? More specifically, if OVX has the strongest impact on sector 1 volatility, does it also have the strongest impact on sector 2 volatility? Moreover, does OVX has significant impact on non-energy-related sector volatility?

In this study, based on the data sample span from Mar 16, 2011 to Dec 31, 2019 and VAR model, we thoroughly examine the impact of OVX on ten sector volatilities. For all sectors, we find the impact of OVX are more economically and statistically significant than realized volatility of both WTI and Brent oil prices, especially after the Chinese refined oil pricing reform of March 27, 2013. We contribute to the existing literatures from three aspects. Firstly, we provide empirical evidences that OVX shows the most influential impact on Chinese stock volatilities. Most researches directly choose OVX, WTI or Brent to embody oil market uncertainty and no comparative analysis are conducted. This study contributes to the choice of OVX from the perspective of volatility spillover from oil market to Chinese stock market. The conclusion highlights the importance of OVX and can provide some meaningful implications. Secondly, this study could be good complements to the existing literatures. For instance, [23,24] study the impact of only OVX on the whole Chinese stock market volatility while we thoroughly investigate the impacts of different oil market uncertainty indictors (OVX, WTI, Brent) on Chinese stock sector (including non-energy-related sectors) volatilities. To some extent, our study provides more comprehensive evidences. Moreover, [25] identify the most influential energy stocks in Chinese stock market and we identify the most influential oil market uncertainty indicators (at least for the response of Chinese stock market volatilities). These two studies seem to be complementary and a further step could be identifying which indicators has the strongest impact on the influential energy stocks. Last but not least, for some researches document the insignificant spillover effects between oil market and stock market, they could revisit by utilizing OVX rather than WTI and/or Brent to embody the oil market uncertainty.

The rest of this paper is scheduled as follows. Section 2 depicts economic models and the data. Section 3 shows the empirical analysis. Section 4 concludes.

## 2. Data and model framework

### 2.1. Data

In this study, OVX, raw WTI and Brent oil prices realized volatility, CSI 300 index (Csi) along with ten Chinese sector indexes are selected to investigate the impact of OVX on Chinese stock markets at volatility level. The sector indexes are Health Care Index (Hea), Information Technology Index (Inf), Consumer Staples Index (Con), Energy Index (Ene), Consumer Discretionary Index (Cod), Financials Index (Fin), Utilities Index (Uti), Industrials Index (Ind), Telecommunication Services Index (Tel) and Materials Index (Mat). Csi was chosen as the proxy for Chinese stock market since the 300 constituents form Csi cover more than half of China’s total equity market capitalization and the constituents of ten sector indexes are selected from Csi. The daily data of OVX, WTI and Brent oil prices, the eleven Chinese stock indexes (all from March 16, 2011 to Dec 31, 2019) are respectively extracted from CBOE and Shanghai Stock Exchange, totally 2053 samples. The sample start from the date of first published VXFXI (control variable) data and end to the date before constructing this study. The plots of all data we are presented in Appendix.

#### 2.1.1. Volatility of sector index

A popular model-free volatility measurement is used to compute the volatility of Chinese sector index:

$${\left(r_{t}\right)}^{2}={\left\{100*\left[\text{ln}\left(p_{t}\right)-\text{ln}\left(p_{t-1}\right)\right]\right\}}^{2}$$

where *p*_*t*_ denotes the closing price of sector index.

#### 2.1.2. Oil market uncertainty

Three indicators are used to embody the oil market uncertainty. Specifically, the commonly used oil market volatility derived from oil prices:

$${\left(or_{t}\right)}^{2}={\left\{100*\left[\text{ln}\left(op_{t}\right)-\text{ln}\left(op_{t-1}\right)\right]\right\}}^{2}$$

where *op*_*t*_ denotes the crude oil daily closing price of WTI or Brent. Another indicator is computed as (*ovx*_*t*_)^2^, where *ovx*_*t*_ denotes daily value of OVX.

#### 2.1.3. Control variables

The Chinese stock market implied volatility index (VXFXI) measure the investor sentiment in Chinese stock market is published by the CBOE in 2011, which is one of the newest implied volatility indices. The construction method of VXFXI is similar with that of OVX and VIX, which imply they have similar intrinsic characteristics and some researches utilize VXFXI to embody the Chinese stock uncertainty [22,23]. Conditional on that, it is reasonable and natural that VXFXI may impact the volatilities of Chinese sector indexes. So (VXFXI)^2^ is chosen as our first control variable.

Given the dominate role of American stock market around the whole world, the volatility spillover from American stock market to Chinese stock market should not be ignored. Moreover, [26] implies VIX is more informative than realized volatility of S&P500 as to the investigations of stock volatility spillover from USA to other countries. Specifically, based on heterogeneous autoregressive (HAR) model, they show VIX information can substantially improve the realized volatility forecasting performance of all 17 global stock indices they selected and the improvements remain statistically significant for horizons up to one mouth. That show strong evidences of volatility spillover from U.S. stock market to other country stock markets. So (VIX)^2^ is chosen as our second control variable. Daily data of VXFXI and VIX (from March 16, 2011 to Dec 31, 2019) are extracted from CBOE.

### 2.2. Model framework

In fact, we are interested in the impact of oil market uncertainty on Chinese stock volatility, while the relationship between them is not unidirectional and the reverse impact may exist either. Moreover, there may exist significant relationship between OVX and VIX (e.g., [27]); OVX and VXFXI (e.g., [23,24]). That result these variables influence each other and vector auto-regression (VAR) model can address the endogenous problems. Hence, we use VAR to analyze the impact of oil market uncertainty on Chinese sector indexes volatilities and VAR model has been considered to be effective for investigating spillover effects between oil and other markets, and many literatures base their researches on that sound model ([21,28–30]; among others). Our model contains four variables: volatility of sector indexes ((*r*_*t*_)^2^), oil market uncertainty and two control variables mentioned before. Oil market uncertainty can be (*ovx*_*t*_)^2^ or the (*or*_*t*_)^2^ of WTI or Brent, that allow us to compare impacts of these three indicators on Chinese sector indexes volatilities. All variables used in this study are tested to be stationary at 95% confidence level. The orders of VAR models are selected by Schwarz Criterion (SC) and the maximum orders are set to 5.

The generalized impulse response functions (girf, the girf does not require orthogonalization of shocks and is invariant to the ordering of the variables in the VAR [31]) derived from VAR estimations are used to analyze the impact of oil market uncertainty on Chinese sector indexes volatilities.

## 3. Empirical analysis

### 3.1. The impact of oil market uncertainty on Chinese sector indexes volatilities

As VAR model require stationarity of variables, we first test whether the variables of interests are stationary. The results based on popular Phillips and Perron (PP) test are shown in Table 1 and the null hypotheses that the variable is not stationary are rejected in all cases. Then we utilize VAR models to analyze the impacts of different oil market uncertainty proxies on Chinese sector volatilities.

**Table 1:** The p values of PP test of all variables.

Control variables	VIX	VXFXI									
PP test	0.010	0.010									
Oil market uncertainty	OVX	WTI	Brent								
PP test	0.010	0.010	0.010								
Sector volatility	Csi	Hea	Inf	Con	Ene	Cod	Fin	Uti	Ind	Tel	Mat
PP test	0.010	0.010	0.010	0.010	0.010	0.010	0.010	0.010	0.010	0.010	0.010

Note that all unit root modules of VAR model should be smaller than one to ensure the impulse responses converge to zero with the increase of the lag order. Table 2 has shown the maximum unit root module of each VAR model and we can find all of them are smaller than one, which imply our models are rational and we can further calculate the impulse response.

**Table 2:** Maximum unit root module of each VAR models.

	Csi	Hea	Inf	Con	Ene	Cod	Fin	Uti	Ind	Tel	Mat
OVX	0.983	0.983	0.978	0.983	0.979	0.983	0.979	0.983	0.983	0.983	0.983
WTI	0.970	0.971	0.953	0.971	0.953	0.971	0.953	0.970	0.970	0.971	0.970
Brent	0.953	0.971	0.953	0.971	0.953	0.971	0.953	0.970	0.970	0.971	0.970

**Fig 1:**
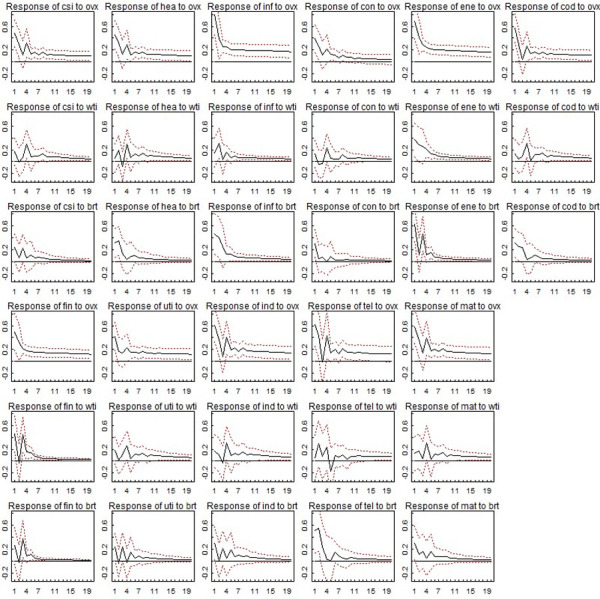
Generalized impulse response of Chinese sector indexes volatilities to three different kinds of oil market uncertainty. Notes: The black lines depict the generalized impulse responses of Chinese stock sector volatilities to one standard deviation shocks of three oil price uncertainty indicators (OVX, WTI, Brent). The red dots depict confidence intervals based on 95% confidence level.

Fig 1 shows the generalized impulse response of Chinese sector indexes volatilities to oil market uncertainty shocks. There are three main findings we can derive from Fig 1. Firstly, at first glance, for all sector indexes we can observe that the black lines (i.e., the response of sectors volatility to oil market uncertainty shocks) lay above zero line in most cases. That means the responses are positive which imply higher oil price uncertainty tends to trigger higher Chinese sector volatilities. This is not surprising and is consistent with previous literatures’ conclusions that there exists volatility spillover from oil market to stock markets. A reason for that is as oil is an important energy source, volatile oil price means volatile cost of goods which further imply fluctuation in corporate earnings, that causes stock price to be more volatile. Secondly, we can find the black lines in the first/fourth row is higher than that in the second and third/fifth and sixth rows. That means the level of responses of indexes volatilities to one standard deviation of OVX shocks seem higher than to one standard deviation of other two commonly used oil market uncertainty indicators (WTI and Brent) shocks. Moreover, the red dots imply OVX shocks effects are more statistically significant than the other two. We do not present the girf results of VIX and VXFXI here (control variables), the estimations of them indicate the impact of VIX and VXFXI on Chinese sector indexes volatilities are statistically significant in most cases. That implies our model framework is reasonable to some extent. Taken together, they show the impact of OVX on Chinese stock market volatility is more economically and statistically significant than that of realized volatility of both WTI and Brent oil prices. This is important, it means when we focus on oil-Chinese stock volatility nexus, OVX seems more informative than WTI and Brent oil price realized volatility. A plausible explanation is OVX contains both historical and future information of oil market while the other two indicators based on raw oil prices contain only historical information, which makes OVX seems more informative than the other two. Thirdly, interestingly, we do not find remarkable evidence that show sector-specific for our conclusions, the result that OVX show the strongest influence among the three oil price uncertainty indicators seems hold for all sectors. That implies the market participants in all sectors can benefit from our conclusion.

It is worth noting that not all the responses of sector indexes volatilities to oil market uncertainty shocks maximize at the beginning. For instance, response of Hea to OVX maximize at first-order lag (row 1, column 2), response of Hea to realized volatility of WTI maximize at fourth-order lag (row 2, column 2), response of Hea to realized volatility of Brent maximize at second-order lag (row 3, column 2). To make the three oil market uncertainty indicators more comparable, we additionally compute their cumulative impulse response.

Table 3 shows the cumulative responses (i.e., cumulative girfs) of sector indexes volatilities to one standard deviation oil market uncertainty shocks. More specifically, for example, one day girf of Csi to OVX (i.e., 0.479) means one standard deviation increases of OVX (i.e., (*ovx*_*t*-1_)^2^ tends to increase Csi volatility (i.e., (*r*_*t*_)^2^) by 0.479 (Mean of Csi volatility is 5.826). The main result is consistent with that of Fig 1, while more visible. On the one hand, the cumulative girfs in OVX column are larger than the corresponding numbers in WTI and Brent column. With the increase of lags, according to Fig 1, the girfs of the Chinese stock volatility to shocks tend to zero which further means the cumulative girfs of Chinese stock volatility to oil market uncertainty shocks tend to be gradually stable. In this sense, for 20 days cumulative responses of the sectors, we can find the responses to OVX shocks are about twice as much as that to WTI and Brent shocks. In another word, for the future Chinese stock sector volatilities, the impacts of one standard deviation OVX changes are about twice as much as that of one standard deviation WTI/Brent changes. That may imply the cumulative responses of Chinese sector volatilities to OVX shocks are more economically significant than that to the other two oil market uncertainty indicators. On the other hand, from the perspective of statistical significance, it is obvious the p-values in OVX column are smaller than that in WTI and Brent column. Moreover, according to the p-values, the cumulative responses of some non-energy related sectors (e.g., Consumer Staples Index (Con); Telecommunication Services Index (Tel)) to WTI and Brent shocks seems to be insignificant while that to OVX shocks are significant. That imply the cumulative girfs of Chinese sector volatilities to OVX shocks are more statistically significant than that to WTI and Brent shocks. Hence, combine the above economic and statistic aspects, we conclude that the cumulative responses of Chinese sector volatilities to OVX shocks are more economically and statistically significant than that to the other two oil market uncertainty indicators shocks. A plausible explanation is OVX contains future information which is not incorporated in WTI and Brent prices, which further makes OVX is relatively more informative.

**Table 3:** Cumulative response of Chinese sector indexes volatilities to three different kinds of oil market uncertainty.

	1 day			5 days			20 days		
	OVX	WTI	Brent	OVX	WTI	Brent	OVX	WTI	Brent
Csi	0.479	0.170	0.230	1.325	0.598	0.669	3.048	1.689	1.164
	(0.000)	(0.150)	(0.051)	(0.000)	(0.075)	(0.049)	(0.000)	(0.023)	(0.096)
Hea	0.452	0.054	0.309	1.301	0.541	0.910	3.136	1.835	1.480
	(0.000)	(0.658)	(0.012)	(0.000)	(0.125)	(0.011)	(0.000)	(0.027)	(0.058)
Inf	0.797	0.206	0.464	1.905	0.986	1.355	4.726	2.358	2.155
	(0.000)	(0.234)	(0.007)	(0.000)	(0.029)	(0.002)	(0.000)	(0.001)	(0.001)
Con	0.389	0.133	0.302	1.107	0.332	0.498	2.009	1.160	0.788
	(0.001)	(0.260)	(0.011)	(0.000)	(0.322)	(0.141)	(0.011)	(0.142)	(0.287)
Ene	0.679	0.365	0.626	1.841	1.248	1.457	4.566	2.261	2.028
	(0.000)	(0.012)	(0.000)	(0.000)	(0.001)	(0.000)	(0.000)	(0.000)	(0.000)
Cod	0.571	0.132	0.307	1.299	0.589	0.884	3.207	1.884	1.458
	(0.000)	(0.285)	(0.012)	(0.000)	(0.090)	(0.012)	(0.000)	(0.020)	(0.057)
Fin	0.499	0.460	0.256	1.372	1.173	0.781	3.416	1.749	1.084
	(0.001)	(0.003)	(0.100)	(0.000)	(0.003)	(0.043)	(0.000)	(0.000)	(0.020)
Uti	0.423	0.180	0.236	1.114	0.601	0.549	3.113	1.898	1.130
	(0.000)	(0.128)	(0.045)	(0.001)	(0.093)	(0.129)	(0.000)	(0.032)	(0.177)
Ind	0.609	0.170	0.304	1.641	0.632	0.753	4.156	2.073	1.475
	(0.000)	(0.231)	(0.032)	(0.000)	(0.134)	(0.077)	(0.000)	(0.047)	(0.136)
Tel	0.625	0.056	0.512	1.610	0.486	1.326	3.761	1.687	2.006
	(0.001)	(0.771)	(0.008)	(0.001)	(0.377)	(0.017)	(0.002)	(0.168)	(0.083)
Mat	0.586	0.125	0.302	1.637	0.674	0.764	3.945	2.029	1.385
	(0.000)	(0.386)	(0.036)	(0.000)	(0.108)	(0.072)	(0.000)	(0.040)	(0.138)

Notes: The numbers outside the parathesis denote the cumulative generalized impulse responses of Chinese stock sector volatilities to one standard deviation shocks of three oil price uncertainty indicators (OVX, WTI, Brent). The numbers in the parathesis denote p-values of null hypothesis that there are no cumulative responses of Chinese sector indexes volatilities to oil market uncertainty shocks.

In summary, with respect to volatility spillover from oil market to Chinese stock market, Fig 1 and Table 3 jointly suggest OVX is more informative than realized volatility of WTI and Brent oil price for all sectors. This conclusion will provide important implications for participants who are concerned about Chinese stock market volatility.

### 3.2. Sub-sample check

Our sample covers the Chinese refined oil pricing reform of March 27, 2013 and that reform are thought as a milestone in the history of oil pricing mechanism of china [13,21]. The reform shortens the oil pricing cycle from 22 workdays to 10 workdays and release the 4% band of oil prices. In a word, this reform greatly relaxes the control of domestic oil prices in China. So we split our sample based on the reform and what counts is whether our conclusions in section 3.1 maintain after the reform. Table 4 shows Cumulative response of Chinese sector indexes volatilities to three different oil market uncertainty indicators before and after the reform. Before the reform, OVX shocks show significant 1-day and 5-day cumulative impacts on three sector indexes (Ene, Uti, Ind), while the impacts of other two oil market uncertainty indicators seem insignificant for all Chinese sector indexes volatilities. Moreover, the girfs of OVX are the largest one among the three indicators in most cases. In a word, Panel A does not contaminate our conclusions. After the reform, the results consolidate that of Table 3, the cumulative responses of all Chinese sector volatilities to OVX shocks are significant and are more economically and statistically significant than other two oil market uncertainty indicators. In summary, the Chinese refined oil pricing reform of March 27, 2013 does not change our conclusions and OVX show the most economically and statistically significant impacts on all Chinese sector indexes volatilities among the three indicators after the reform. From Panel A and B, we could make a speculation that the oil pricing reform enhances the volatility spillover effects as Panel B shows more significant results than Panel A. Possible explanation is the reform release the control of domestic oil price and this allows changes in domestic oil prices to more promptly catch up with changes in international oil prices, which further result the stronger reaction of domestic stock market volatility to international oil market volatility.

**Table 4:** Cumulative response of Chinese sector indexes volatilities to three different kinds of oil market uncertainty (before and after the reform).

	1 day			5 days			20 days		
	OVX	WTI	Brent	OVX	WTI	Brent	OVX	WTI	Brent
	Panel A: Before the reform (from March 16, 2011 to March 26, 2013)								
Csi	0.227	0.103	0.027	0.225	0.155	0.174	0.305	0.245	0.221
	(0.109)	(0.469)	(0.852)	(0.376)	(0.539)	(0.473)	(0.533)	(0.397)	(0.402)
Hea	0.053	0.044	0.064	0.040	0.110	0.144	-0.107	0.027	0.089
	(0.713)	(0.763)	(0.658)	(0.881)	(0.684)	(0.580)	(0.833)	(0.928)	(0.746)
Inf	0.309	-0.008	0.031	0.459	0.279	0.509	0.631	0.324	0.486
	(0.121)	(0.969)	(0.877)	(0.218)	(0.453)	(0.157)	(0.387)	(0.455)	(0.221)
Con	0.080	0.048	0.101	0.190	0.051	0.040	0.178	0.013	0.001
	(0.561)	(0.726)	(0.464)	(0.445)	(0.836)	(0.866)	(0.708)	(0.962)	(0.997)
Ene	0.471	0.105	-0.019	0.834	0.578	0.387	1.308	0.686	0.415
	(0.030)	(0.630)	(0.929)	(0.035)	(0.141)	(0.305)	(0.090)	(0.133)	(0.321)
Cod	0.216	0.038	-0.011	0.309	0.056	0.108	0.469	0.134	0.128
	(0.129)	(0.789)	(0.940)	(0.218)	(0.823)	(0.655)	(0.338)	(0.646)	(0.633)
Fin	-0.020	0.060	-0.016	-0.693	-0.045	0.002	-1.259	0.023	0.070
	(0.923)	(0.769)	(0.936)	(0.082)	(0.911)	(0.997)	(0.117)	(0.959)	(0.865)
Uti	0.379	0.162	0.125	0.569	0.337	0.405	0.920	0.480	0.502
	(0.003)	(0.199)	(0.320)	(0.022)	(0.171)	(0.092)	(0.063)	(0.101)	(0.064)
Ind	0.433	0.152	0.040	0.560	0.304	0.354	0.783	0.407	0.402
	(0.008)	(0.353)	(0.807)	(0.050)	(0.281)	(0.191)	(0.155)	(0.214)	(0.179)
Tel	0.284	-0.098	-0.023	0.476	-0.038	0.221	0.539	-0.006	0.216
	(0.128)	(0.598)	(0.904)	(0.142)	(0.906)	(0.473)	(0.378)	(0.986)	(0.515)
Mat	0.346	-0.001	-0.039	0.738	0.245	0.192	0.983	0.258	0.152
	(0.088)	(0.995)	(0.848)	(0.059)	(0.533)	(0.612)	(0.200)	(0.573)	(0.715)
	Panel B: After the reform (from March 26, 2013 to Dec 31, 2019)								
Csi	0.676	0.231	0.196	1.466	0.650	0.366	3.620	1.931	1.055
	(0.000)	(0.117)	(0.192)	(0.000)	(0.093)	(0.201)	(0.000)	(0.002)	(0.024)
Hea	0.628	0.075	0.312	1.853	0.484	0.917	3.974	1.994	1.662
	(0.000)	(0.627)	(0.043)	(0.000)	(0.239)	(0.024)	(0.000)	(0.004)	(0.011)
Inf	0.995	0.041	0.307	2.079	0.471	0.775	4.952	1.830	1.855
	(0.000)	(0.848)	(0.153)	(0.000)	(0.264)	(0.060)	(0.000)	(0.011)	(0.008)
Con	0.521	0.160	0.211	0.935	0.375	0.341	1.965	1.278	0.912
	(0.001)	(0.276)	(0.174)	(0.000)	(0.375)	(0.243)	(0.011)	(0.187)	(0.036)
Ene	0.790	0.371	0.679	2.198	1.157	1.270	4.926	2.722	2.010
	(0.000)	(0.032)	(0.000)	(0.000)	(0.011)	(0.005)	(0.000)	(0.000)	(0.004)
Cod	0.834	0.197	0.275	1.628	0.709	0.721	3.877	2.215	1.480
	(0.000)	(0.206)	(0.082)	(0.000)	(0.079)	(0.016)	(0.000)	(0.001)	(0.004)
Fin	0.678	0.437	0.143	1.594	0.493	0.056	3.982	1.177	0.626
	(0.000)	(0.022)	(0.455)	(0.000)	(0.172)	(0.873)	(0.000)	(0.017)	(0.188)
Uti	0.510	0.208	0.254	1.541	0.718	0.605	4.077	2.225	1.396
	(0.001)	(0.165)	(0.090)	(0.000)	(0.096)	(0.158)	(0.000)	(0.002)	(0.044)
Ind	0.718	0.215	0.356	2.304	0.785	0.777	5.325	2.588	1.698
	(0.000)	(0.230)	(0.047)	(0.000)	(0.123)	(0.123)	(0.000)	(0.003)	(0.039)
Tel	0.931	0.030	0.500	1.891	0.651	1.344	4.345	2.022	2.426
	(0.000)	(0.905)	(0.045)	(0.000)	(0.194)	(0.006)	(0.001)	(0.011)	(0.002)
Mat	0.855	0.178	0.269	1.906	0.861	0.572	4.726	2.663	1.499
	(0.000)	(0.320)	(0.141)	(0.000)	(0.077)	(0.115)	(0.000)	(0.001)	(0.014)

Notes: The numbers outside the parathesis denote the cumulative generalized impulse responses of Chinese stock sector volatilities to one standard deviation shocks of three oil price uncertainty indicators (OVX, WTI, Brent). The numbers in the parathesis denote p-values of null hypothesis that there are no cumulative responses of Chinese sector indexes volatilities to oil market uncertainty shocks.

There may exist some potential inconsistences between this study and existing literatures. For the volatility spillover from oil market to Chinese stock market, some researches provide the evidences of insignificant effects. For example, the work by [32] employ Multiple-GARCH models to investigate the volatility spillover between oil market and three Asian-importing countries (Chinese, Japanese and Indian) stock markets. Based on daily WTI oil price data, they fail to find significant spillover between oil market and Chinese stock market. The work of [33] investigate the volatility spillover among crude oil, natural gas, coal, stock, and currency markets in the US and China, conditional on the volatility spillover index of [34]. By using daily WTI data, they show although volatility spillovers of WTI significantly affect those of the S&P500, Chinese stock market volatility seems not be influenced by WTI. By employing similar method, [35] examines the volatility transmission between WTI and stock markets of G7 countries plus India and China. They find the significant volatility spillover amid stock markets, while no significant evidences of volatility transmission between WTI and stock markets are found. The aforementioned literatures base their study on WTI crude oil prices and opine volatility of oil market do not affect Chinese stock volatility. In this study, we can find the impacts of OVX on Chinese stock volatilities are highly significant, especially after the reform, and the OVX’s impacts are more significant than other two crude oil prices’ impacts. That imply using different oil market volatility indictors may lead to different conclusions. Moreover, from the perspective of stock sectors, some researches show the volatility spillover between oil market and stock sectors are sector-specific. More specifically, their studies show volatilities of some sectors response to oil volatility significantly, while the others not. For example, [36] deploy bivariate GARCH models to explore the volatility transmission between oil price and five U.S. sectors. Based on weekly data of WTI prices, they find oil volatility significantly affect the volatilities of financial sector, technology sector and consumer sector, while that effects for health and industrial sectors are insignificant. Based on daily Brent oil prices, [37] related to Indian sectors and [38] related to Eurozone sectors derive the similar conclusions that oil volatility influence volatilities of some sectors while others not. In this study, we provide evidences that OVX show significant impacts on all sector volatilities in Chinese stock market while the impacts of WTI and Brent are limited to certain sectors. That may imply OVX is more informative than crude oil prices with respective to oil-stock volatility connectiveness. In summary, we try to compare the potential inconsistence between our results and existing literatures from the perspectives of aggregate level and sector level. The comparations highlight the importance of OVX in terms of volatility transmission between oil market and stock markets.

### 3.3. Robust test by using the volatility estimated from the GARCH-family model

We conduct a robustness check by using the conditional volatility estimated from the GARCH-family model. Specifically, for WTI and Brent volatility and Chinese stock sectors volatilities, we replace the square of log returns with the volatilities estimated from GARCH models.

At first, we obtain the volatilities of WTI, Brent and 11 Chinese sectors from ARMA (1,1)-GARCH (1,1) model and repeat the analysis process of section 3.1 and 3.2. Similarly, we check whether these volatility series are stationary based on PP test and the results are presented in Table 5. The results imply the most variables seem to be non-stationary. Accordingly, we take first difference for all series (including OVX and controlling variables) and implement our analysis based on the new series. The p values of PP test of new series are presented in Table 6 and we can find all of them become stationary.

**Table 5:** The p values of PP test of WTI, Brent and 11 Chinese sector volatilities estimated from GARCH models.

	WTI	Brent	Csi	Hea	Inf	Con	Ene	Cod	Fin	Uti	Ind	Tel	Mat
PP test	0.016	0.042	0.07	0.207	0.371	0.034	0.015	0.191	0.019	0.207	0.191	0.049	0.041

**Table 6:** The p values of PP test of all new variable series.

Control variables	VIX	VXFXI									
PP test	0.010	0.010									
Oil market uncertainty	OVX	WTI	Brent								
PP test	0.010	0.010	0.010								
Sector volatility	Csi	Hea	Inf	Con	Ene	Cod	Fin	Uti	Ind	Tel	Mat
PP test	0.010	0.010	0.010	0.010	0.010	0.010	0.010	0.010	0.010	0.010	0.010

Subsequently, we estimate the VAR models (orders are selected by Schwarz Criterion (SC) and the maximum orders are set to 5). Similar to Tables 2 and 7 presents the maximum unit root module for each estimated VAR models and we can find all of them are smaller than one. That imply our models are rational and we can further analyze the generalized impulse responses of Chinese sector volatilities to three different oil market uncertainty proxy shocks.

**Table 7:** Maximum unit root module of each VAR models (GARCH volatilities).

	Csi	Hea	Inf	Con	Ene	Cod	Fin	Uti	Ind	Tel	Mat
OVX	0.573	0.593	0.58	0.571	0.587	0.594	0.398	0.581	0.606	0.565	0.578
WTI	0.588	0.602	0.279	0.279	0.39	0.611	0.283	0.382	0.615	0.279	0.594
Brent	0.404	0.591	0.28	0.28	0.401	0.596	0.284	0.395	0.609	0.28	0.397

**Fig 2:**
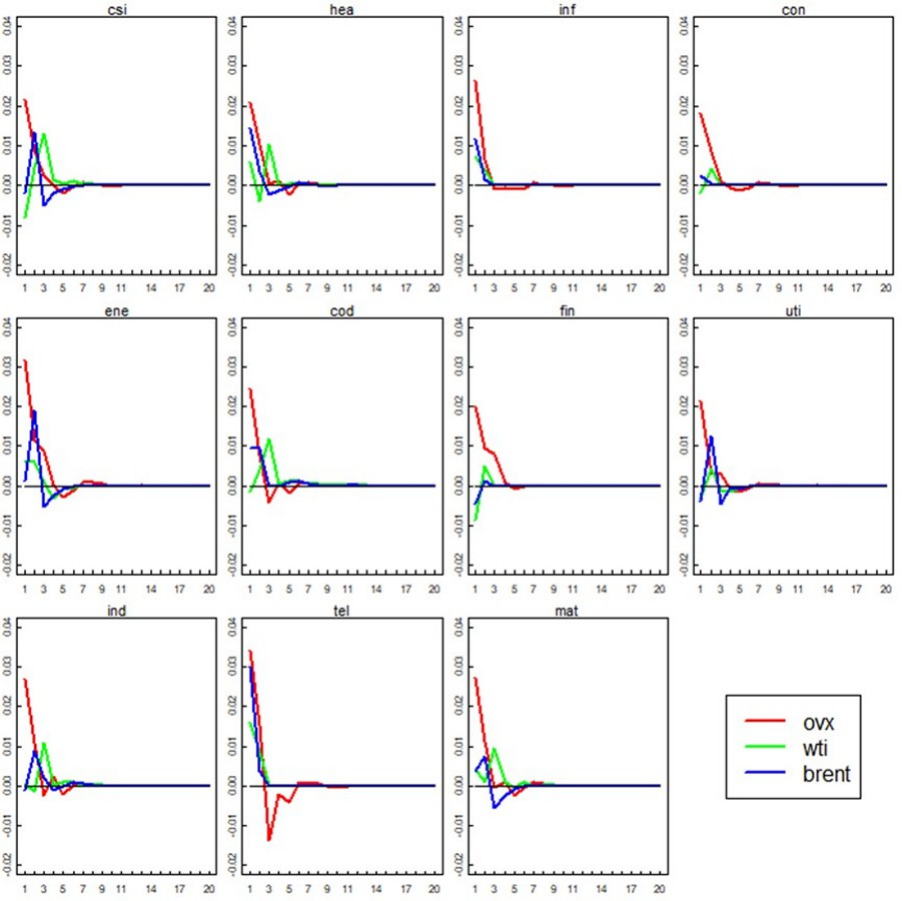
Generalized impulse response of Chinese sector indexes volatilities to three different kinds of oil market uncertainty (GARCH volatilities).

Fig 2 show the detailed results of generalized impulse responses. The red, green and blue line indicate the response of Chinese sector volatilities to OVX, WTI and Brent shocks, respectively. It is obvious the red lines lay above the green and blue lines at the beginning for all cases, which indicates the impact of previous days’ OVX on sector volatilities is larger than that of previous days’ WTI and Brent. Also, not all impulse responses maximize at the beginning. Generally, the results derived from Fig 2 are similar to that derived from Fig 1.

We further calculate the cumulative impulse responses to make the comparation more comprehensive and intuitive. Table 8 depict the detailed results and we can find the cumulative responses of sector volatilities to OVX are larger than that to WTI and Brent for almost all cases (except for “tel” (5 days and 20 days)). In summary, the results of Table 8 are consistent with that of Table 3.

**Table 8:** Cumulative response of Chinese sector indexes volatilities to three different kinds of oil market uncertainty (GARCH volatilities).

		1 day			5 days			20 days	
	OVX	WTI	Brent	OVX	WTI	Brent	OVX	WTI	Brent
Csi	0.022	-0.008	-0.002	0.031	0.011	0.004	0.032	0.014	0.004
Hea	0.021	0.006	0.014	0.031	0.012	0.014	0.032	0.014	0.015
Inf	0.026	0.007	0.012	0.03	0.011	0.013	0.031	0.011	0.013
Con	0.018	-0.002	0.003	0.026	0.002	0.003	0.026	0.002	0.003
Ene	0.032	0.006	0.001	0.049	0.01	0.012	0.05	0.009	0.011
Cod	0.025	-0.001	0.009	0.027	0.016	0.019	0.029	0.019	0.022
Fin	0.02	-0.009	-0.005	0.038	-0.004	-0.004	0.037	-0.004	-0.004
Uti	0.021	-0.003	-0.004	0.025	-0.002	0.003	0.026	-0.003	0.003
Ind	0.027	0.001	-0.001	0.036	0.011	0.009	0.037	0.013	0.011
Tel	0.034	0.016	0.03	0.029	0.025	0.034	0.031	0.025	0.034
Mat	0.027	0.004	0.004	0.037	0.016	0.002	0.038	0.017	0.002

At last, we split our sample based on the Chinese refined oil pricing reform of March 27, 2013 and repeat the previous process, which could check whether the reform impacts our conclusion. To save space, we only present the cumulative impulse responses before and after the reform and the detailed results are presented in Table 9. Two main findings could be derived from Table 9. Firstly, no matter for the sample before or after the reform, the responses of sectors to OVX are larger than that to WTI and Brent for most cases. This is consistent with our main conclusion. Secondly, the responses after the reform seem larger than that before the reform, which may imply the reform enhances the volatility spillover effect from oil market to Chinese stock market. In summary, the results of Table 9 are consistent with that of Table 4.

**Table 9:** Cumulative response of Chinese sector indexes GARCH volatilities to three different kinds of oil market uncertainty (before and after the reform).

		1 day			5 days			20 days	
	OVX	WTI	Brent	OVX	WTI	Brent	OVX	WTI	Brent
Panel A: Before the reform (from March 16, 2011 to March 26, 2013)									
Csi	0.013	-0.001	0.007	0.016	0.000	0.007	0.017	0.000	0.007
Hea	0.005	0.001	0.002	0.007	0.002	0.001	0.008	0.002	0.001
Inf	0.012	0.004	0.015	0.012	0.006	0.014	0.013	0.006	0.014
Con	0.006	-0.002	-0.001	0.010	-0.001	-0.001	0.011	-0.001	-0.001
Ene	0.022	0.023	0.024	0.021	0.026	0.023	0.021	0.026	0.023
Cod	0.011	-0.002	0.009	0.012	0.000	0.008	0.013	0.000	0.008
Fin	0.009	-0.004	0.008	0.012	-0.003	0.008	0.012	-0.003	0.008
Uti	0.022	-0.003	0.010	0.025	0.002	0.007	0.026	0.002	0.007
Ind	0.021	0.002	0.015	0.021	0.004	0.014	0.022	0.004	0.014
Tel	0.018	0.005	0.018	0.025	0.006	0.016	0.025	0.007	0.016
Mat	0.016	0.005	0.012	0.022	0.007	0.011	0.023	0.007	0.011
Panel B: After the reform (from March 26, 2013 to Dec 31, 2019)									
Csi	0.028	-0.006	-0.003	0.054	-0.001	-0.004	0.057	-0.001	-0.003
Hea	0.029	0.011	0.018	0.052	0.005	0.011	0.056	0.004	0.011
Inf	0.036	0.008	0.012	0.039	0.015	0.013	0.039	0.015	0.013
Con	0.025	-0.002	0.004	0.028	0.005	0.002	0.028	0.005	0.002
Ene	0.039	0.006	-0.002	0.076	0.012	0.002	0.080	0.012	0.002
Cod	0.033	0.002	0.011	0.049	0.007	0.014	0.053	0.007	0.014
Fin	0.030	-0.010	-0.006	0.036	0.001	-0.008	0.036	0.001	-0.008
Uti	0.024	-0.001	-0.003	0.036	0.005	-0.004	0.038	0.005	-0.004
Ind	0.031	0.005	-0.003	0.056	0.003	-0.003	0.061	0.003	-0.003
Tel	0.050	0.019	0.033	0.057	0.033	0.035	0.057	0.033	0.035
Mat	0.035	0.010	0.005	0.065	0.021	0.006	0.069	0.021	0.006

All in all, in section 3.3, we conduct a robust check of by replacing the square log returns with corresponding volatilities derived from GARCH models. The results of section 3.3 cannot overturn our conclusion that the impacts of OVX on Chinese sector volatilities are more significant than that of WTI and Brent.

## 4. Conclusion

This study examines the impact of three different oil market uncertainty indicators on Chinese sector indexes volatilities. The full-sample and sub-sample empirical results could come to a conclusion. The impact of OVX on Chinese sector volatility is significant and are more economically and statistically significant than traditional realized volatility of both WTI and Brent oil prices, especially after the Chinese refined oil pricing reform of March 27, 2013 and, that holds true for all sectors.

This study could provide some meaningful implications for market regulators and investors. For instance, our results imply OVX contain more information than realized volatility of both WTI and Brent raw oil prices and thus the regulators should concern not only WTI and Brent oil prices but also OVX. Meanwhile, policymakers are suggested to improve the mechanisms to weaken the potential negative impacts from OVX shocks due to the significant impact of OVX on all sectors. More importantly, policy makers should adopt appropriate policies to guide investors to reduce panic when faced with the similar reform which loosen the control of domestic oil price, as that reform tends to intensify the volatility (risk) spillover from oil market to Chinese stock market. For the investors in any sectors of Chinese stock market, OVX should attract more attention than traditional raw oil price series as it may offer additional significant information for volatility forecasting which is vital to investors. They can try to introduce OVX instead of realized volatility of WTI and Brent into their models, which may produce more desired results for rebalancing and adjusting their positions and strategy. The last but not least, for the scholars, compared with the traditional oil price series, the research on oil-stock nexus from the perspective of OVX may produce different findings. This paper provides empirical evidences for that and thus our study enriches the understanding of oil-stock market nexus. It is worth noting that, from the perspective of market participants, it would be of practical importance either to understand the economic significance of each proxy in shaping their portfolios. The related work would be an interesting point in the future. Another important extension could be the asymmetric and dynamic effects of oil market uncertainty on Chinese stock market volatilities.

## Supporting information

S1 Appendix (DOCX)

S1 File (XLSX)

## References

[1] Ji Q., Bouri E., Roubaud D., 2018. Dynamic network of implied volatility transmission among US equities, strategic commodities, and BRICS equities. International Review of Financial Analysis, Vol. 57, pp. 1–12.

[2] Lin B.Q., Chen Y.F., 2019. Dynamic linkages and spillover effects between CET market, coal market and stock market of new energy companies: A case of Beijing CET market in China. Energy, 172, 1198–1210.

[3] Wen F.H., Cao J.H., Liu Z., Wang X., 2021. Dynamic volatility spillovers and investment strategies between the Chinese stock market and commodity markets. International Review of Financial Analysis, 76, 101772.

[4] Dai Z.F., Luo Z., Liu C., 2023. Dynamic volatility spillovers and investment strategies between crude oil, new energy, and resource related sectors. Resources Policy, Volume 83, 10.1016/j.resourpol.2023.103681.

[5] Iqbal N., Bouri E., Liu G., & Kumar A., 2022. Volatility spillovers during normal and high volatility states and their driving factors: A cross-country and cross-asset analysis. International Journal of Finance & Economics, 1–21. 10.1002/ijfe.2717.

[6] Bouri E., Lei X.J., Jalkh N., Xu Y.H., Zhang H.W., 2021. Spillovers in higher moments and jumps across US stock and strategic commodity markets. Resources Policy, Vol. 72, 102060.

[7] Cong R.G., Wei Y.M., Jiao J.L., Fan Y., 2008. Relationships between oil price shocks and stock market: An empirical analysis from China. Energy Policy. 36, 3544–3553.

[8] Broadstock D.C., Cao H., Zhang D.Y., 2012. Oil shocks and their impact on energy related stocks in China. Energy Economics 34(6), 1888–1895.

[9] Fang C.R., You S.Y., 2014. The impact of oil price shocks on the large emerging countries’ stock prices: Evidence from China, India and Russia. International Review of Economics & Finance 29, 330–338.

[10] Caporale G.M., Ali F.M., Spagnolo N., 2015. Oil price uncertainty and sectoral stock returns in China: A time-varying approach. China Economic Review. 34, 311–321.

[11] Wei Y.F., Guo X.Y., 2017. Oil price shocks and China’s stock market. Energy 140, 185–197.

[12] Hu C.Y., Liu X.H., Pan B., Chen B., Xia XH., 2018. Asymmetric Impact of Oil Price Shock on Stock Market in China: A Combination Analysis Based on SVAR Model and NARDL Model. Emerging markets finance and trade. 54(8), 1693–1705.

[13] Bouri E., Qian C., Lien D., 2017. Causality between oil prices and the stock market in China: The relevance of the reformed oil product pricing mechanism. International Review of Economics & Finance 48, 34–48.

[14] Wang X.X., Wang Y.D., 2019. Volatility spillovers between crude oil and Chinese sectoral equity markets: Evidence from a frequency dynamics perspective. Energy Economics. 80, 995–1009.

[15] Yu L., Zha R., Stafylas D., He K., Liu J., 2020. Dependences and volatility spillovers between the oil and stock markets: New evidence from the copula and VAR-BEKK-GARCH models. International Review of Financial Analysis. 68.

[16] Ahmed A.D., Huo R., 2021. Volatility transmissions across international oil market, commodity futures and stock markets: Empirical evidence from China. Energy Economics. Volume 93, 104741.

[17] Salisu A., Gupta R., Bouri E., Ji Q., 2021. Mixed-Frequency Forecasting of crude oil volatility based on the information content of global economic conditions. Journal of Forecasting, Vol. 44 No. 1, pp. 134–157

[18] Zhang H.W., Jin C., Bouri E., Gao W., Xu Y.H., 2023. Realized higher-order moments spillovers between commodity and stock markets: Evidence from China. Journal of Commodity Markets, Vol. 30, 30, 100275.

[19] Dai Z.F., Zhu H.Y., 2023. Dynamic risk spillover among crude oil, economic policy uncertainty and Chinese financial sectors. International Review of Economics & Finance, Volume 83, Pages 421–450.

[20] Liu M.L., Ji Q., Fan Y., 2013. How does oil market uncertainty interact with other markets? An empirical analysis of implied volatility index. Energy. 55(1), 860–868.

[21] Luo X.G., Qin S.H., 2017. Oil price uncertainty and Chinese stock returns: New evidence from the oil volatility index. Finance Research Letters. 20, 29–34.

[22] Xiao J.H., Zhou M., Wen F.M., Wen F.H., 2018. Asymmetric impacts of oil price uncertainty on Chinese stock returns under different market conditions: Evidence from oil volatility index. Energy Economics. 74, 777–786.

[23] Xiao J.H., Hu C.H., Ouyang G.D., Wen F.H., 2019. Impacts of oil implied volatility shocks on stock implied volatility in China: Empirical evidence from a quantile regression approach. Energy Economics. 80, 297–309.

[24] Chen L., Wen F.H., Li W.Y., Yin H., Zhao L.L., 2022. Extreme risk spillover of the oil, exchange rate to Chinese stock market: Evidence from implied volatility indexes. Energy Economics, 107.

[25] Wang Z., Gao X.Y., An H.Z., Tang R.W., Sun Q.R., 2020. Identifying influential energy stocks based on spillover network. International Review of Financial Analysis, 68, 101277

[26] Daniel Buncic., Gisler Katja I.M., 2016. Global equity market volatility spillovers: a broader role for the United States. International Journal of Forecasting, 32 (4), 1317–1339.

[27] Li Leon, 2022. The dynamic interrelations of oil-equity implied volatility indexes under low and high volatility-of-volatility risk. Energy Economics, Volume 105, 10.1016/j.eneco.2021.105756.

[28] Jin X.J., Zhu F.F., 2019. Global Oil Shocks and China’s Commodity Markets: The Role of OVX, Emerging Markets Finance and Trade, doi: 10.1080/1540496X.2019.1658075

[29] Armeanu D.Ş., Joldeş C.C., Gherghina Ş.C., 2019. On the Linkage between the Energy Market and Stock Returns: Evidence from Romania. Energies, 12, 1463. 10.3390/en12081463.

[30] Khan M. H., Ahmed J., Mughal M., & Khan I. H., 2022. Oil price volatility and stock returns: Evidence from three oil-price wars. International Journal of Finance & Economics, 1–21. 10.1002/ijfe.2588.

[31] Pesaran H.H., Shin Yongcheol., 1998. Generalized impulse response analysis in linear multivariate models. Economics Letters. 58, 17–29.

[32] Sarwar S., Khalfaoui R., Waheed R., Dastgerdi H.G., 2019. Volatility spillovers and hedging: Evidence from Asian oil-importing countries. Resources Policy, Volume 61, Pages 479–488

[33] Asadi M., Roubaud D., Tiwari A.K, 2022. Volatility spillovers amid crude oil, natural gas, coal, stock, and currency markets in the US and China based on time and frequency domain connectedness. Energy Economics, Volume 109, 105961, 10.1016/j.eneco.2022.105961.

[34] Diebold, Francis X., Yilmaz, Kamil, 2012. Better to give than to receive: Predictive directional measurement of volatility spillovers. International Journal of Forecasting 28, 57–66.

[35] Guru B.K., Pradhan A.K., Bandaru R., 2023. Volatility contagion between oil and the stock markets of G7 countries plus India and China. Resources Policy, Volume 81, 103377, 10.1016/j.resourpol.2023.103377.

[36] Malik F., Ewing B.T., 2009. Volatility transmission between oil prices and equity sector returns. International Review of Financial Analysis, Volume 18, Issue 3, Pages 95–100.

[37] Singhal S., Ghosh S., 2016. Returns and volatility linkages between international crude oil price, metal and other stock indices in India: Evidence from VAR-DCC-GARCH models. Resources Policy, Volume 50, Pages 276–288

[38] Belhassine O., 2020. Volatility spillovers and hedging effectiveness between the oil market and Eurozone sectors: A tale of two crises. Research in International Business and Finance, Volume 53, 101195, 10.1016/j.ribaf.2020.101195.

