# Supplementary material for: WTI, Brent or implied volatility index: Perspective of volatility spillover from oil market to Chinese stock market
Source: PLoS One. 2024 Apr 25;19(4):e0302131. doi: 10.1371/journal.pone.0302131 (PMC11045093; doi:10.1371/journal.pone.0302131)
Supplement: S1 Appendix — (DOCX) [file pone.0302131.s001.docx]

**Appendix**

This Appendix provide the plot of realized volatility of WTI, Brent oil prices, all 11 Chinese stock indices in Figure A1. The square of volatility indices namely OVX, VIX and VXFXI are shown in Figure A2.


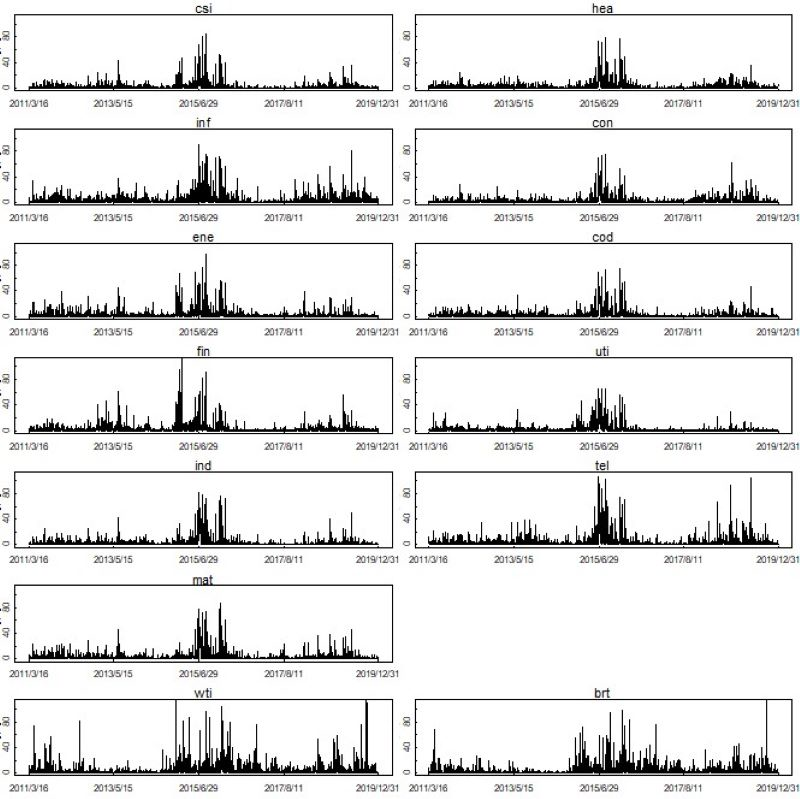


**Figure A1. The realized volatility of all 11 Chinese stock indices as well as that of WTI, Brent oil prices**


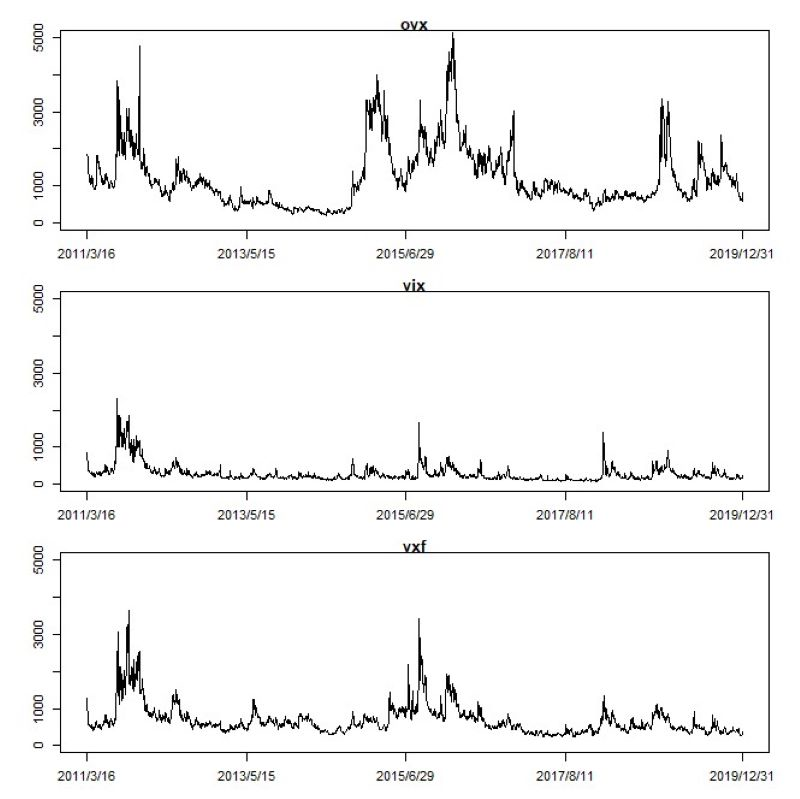


**Figure A2. The square of volatility indices namely OVX, VIX and VXFXI**
